# Are views towards egg farming associated with Brazilian and Chilean egg consumers’ purchasing habits?

**DOI:** 10.1371/journal.pone.0203867

**Published:** 2018-09-28

**Authors:** Dayane Lemos Teixeira, Rafael Larraín, Maria José Hötzel

**Affiliations:** 1 Departamento de Ciencias Animales, Facultad de Agronomía e Ingeniería Forestal, Pontificia Universidad Católica de Chile, Santiago, Chile; 2 Laboratório de Etologia Aplicada, Departamento de Zootecnia e Desenvolvimento Rural, Universidade Federal de Santa Catarina, Florianópolis, Brazil; Tokat Gaziosmanpasa University, TURKEY

## Abstract

In many industrialised countries, public rejection of intensive animal production systems has led to the development of legislation and industry actions that have resulted in significant changes in animal care at the farm level. However, little is known about the views of citizens from emerging countries regarding animal production. The aims of this study were to explore the views of Brazilian and Chilean consumers towards egg farming, and to investigate if these views are associated with participants’ eggs purchasing habits and reported willingness to pay (WTP) more for eggs produced in the conditions they perceive as important. In an open question, participants (n = 716) were asked to describe an ideal egg production farm and explain their reasons. This was followed by closed questions asking egg purchasing habits, willingness to pay for eggs produced in the conditions they perceive as important and demographic information. Participants main concerns were with animal welfare, naturalness, hygiene, production, and ethical aspects, which many associated with improved health, sensory, and nutritional quality of the eggs. The views of participants towards an ideal egg production farm were associated, to some extent, with type of egg purchasing habits and WTP a premium for organic or free-range eggs. Our results suggest a demand for more natural, animal friendly egg production systems; furthermore, they indicate a disconnect between lay citizens’ expectations and industry practices, given that intensive confined systems typically fail to supply many of the expected characteristics.

## Introduction

Animal agriculture has undergone significant changes in the past decades. During the post-war period a series of social changes, including population growth and urbanization, and newly developed technologies created a suitable environment for the intensification of agriculture, which in animal production led to the fast adoption of close confinement and caged housing [1]. Conventional cages became standard housing for laying hens in commercial production systems. In this type of housing, hens do not have sufficient space to walk, stretch, flap their wings or express basic natural behaviours such as nesting or dust bathing [2, 3]. Public rejection to this and other aspects of intensive animal production systems led to the development of legislation and industry actions that have resulted in significant changes in animal care at the farm level [2, 4, 5]. In the case of egg laying hens, conventional cages have been banned in many parts of the world [2, 6].

Egg consumption in Brazil (192 eggs per person/year; [7]) and Chile (200 eggs per person/year; [8]) is slightly lower than in the US, the world leader with 248.5 per person/year [9], and equivalent to places such as The Netherlands (186 eggs per person/year) and the United Kingdom (191 eggs per person/year) [8]. The majority of eggs in Brazil and Chile are still produced in conventional cage systems [10, 11]. The interest of consumers in eggs produced in non conventional systems in Brazil is reflected in legislation regulating organic and free-range egg production [12]; the Chilean association of egg producers, identifying a national demand for eggs produced in cage-free systems, has urged a national regulation to certify such production [13]. In Brazil and Chile some indication of change in the egg industry can be observed, especially coming from multinational processors and retailers. For example, McDonalds (in Brazil), Heins (in Brazil) and Unilever (in both countries) have announced commitments to purchase only eggs produced in cage-free systems within the next decade [14]. The Humane Society International (HIS), one of the largest ONGs in the world engaged in animal protection, launched a campaign to ban conventional cages for laying hens in Brazil [15].

Understanding the values and attitudes of the general public is considered central in the development of sustainable food animal agriculture (e.g., [16]). Despite the fact that the growth in production and demand for animal food products is concentrated in emerging countries, and considering the economic and social relevance of animal agriculture for these nations, little is known about the views of their citizens regarding animal production [5, 17]. It is reported that citizens from Brazil and Chile are interested in the quality of food and in some aspects of food production [18, 19]. However, it appears that this interest is mostly related to aspects of food production that may influence human health and food prices [20, 21], whereas concerns for farm animal welfare seems low [5, 22], which suggests a lack of knowledge of how animals are produced [23, 24]. Furthermore, it has been shown that attitudes of citizens towards production systems are not always reflected in their purchasing habits or willingness to pay more for the attributes they consider important in food production [25]. This issue, however, is also underexplored in the emerging economies [18, 26].

The aims of this study were to explore the views of Brazilian and Chilean consumers towards egg farming, and to investigate if these views are associated with participants’ eggs purchasing habits and stated willingness to pay (WTP) more for eggs produced in the conditions they perceive as important.

## Materials and methods

### Survey content

A convenience sample of participants from Brazil (BR) and Chile (CH) were invited to respond to two open questions: “What do you consider to be an ideal egg production farm?” and “Why do you consider these characteristics important?”. These questions were preceded by multiple-choice demographic questions covering sex, age, level of completed education, area of residence, involvement in animal production, consumption of eggs, milk, pork, chicken meat and beef, and their self-evaluation of knowledge about egg production. Finally, three multiple-choice questions were included: (1) “which is the most important aspects in an egg production farm?” (with options: that it is the best for my health and of my family; that it is the best for the welfare of laying hens; that it is the best for the environment; that it is the best for producing cheaper eggs; other reasons); (2) “which type of eggs do you usually buy?” (with options: regular; free-range; organic; all types in different occasions; I do not buy eggs); and (3) “in comparison with the price that you usually pay, how much would you be willing to pay more to buy eggs produced in the conditions that you described as important for you?” (with options: the same, 5 to 10%; 11 to 35%; double; I do not know; I do not buy eggs). The identity of all participants was kept confidential.

### Sample recruitment

The study was approved by the Humane Research Ethics Board of the Universidade Federal de Santa Catarina (No. 1.386.798). The Research Ethics and Safety Board at Pontificia Universidad Católica de Chile approved the study and granted a Certificate of Exemption (No. 151126003) for the need of consent form, due to the type of questions and the anonymity of the participants. In total, 358 Brazilian (BR) and 358 Chilean (CH) egg consumers answered the survey. Data were collected from September 21, 2015 to November 25, 2015 in Brazil, and from February 03, 2016 to March 22, 2016 in Chile.

We aimed to include a diverse range of participants for this survey while reaching different types of egg consumers, within a public not associated with egg production or agriculture. A convenience sample of participants was recruited both online (including e-mail, social media) and face-to-face. In both cases (personal or online recruitment) the questionnaires were self-administered, with no interaction between recruiter and respondent after the acceptance to participate. The link to access the Google Form (from Google Drive platform) with the questionnaire were sent to e-mail lists of different organizations (e.g. universities, hospital and retirees’ associations), social media outlets such as Facebook and Twitter, targeting groups with food or lifestyle focus, science focus (i.e. science communication, higher education) or current event focus, that operated across different parts of Brazil and Chile. Personal invitations (BR: 71%; CH: 53%) were made in public places (airport and bus station waiting areas and shopping malls) by two researchers in each country. Participants were asked to participate in a survey about animal rearing systems conducted by university researchers and given the following information at the beginning of the survey: “In this study we want to know what you consider important in egg production systems”. No mention to the term animal welfare was made in the questionnaire or in the consent form. No participant declined to answer the survey after start it.

### Identification of themes

Data personally collected were transcribed to the Google Forms used to the online questionnaire. All information was automatically transcribed to a Microsoft Excel (version 2013) sheet. The analysis of the open-ended responses was based on the methodology described by Huberman and Miles [27]. Firstly, themes were identified by coding information (data reduction); secondly, information was organized allowing for conclusions to be drawn (data display); finally, patterns and themes were observed and confirmatory tactics were used (conclusion drawing and verification). Initially, three trained evaluators fluent both in Portuguese and Spanish independently examined 50 randomly selected responses for the Brazilian and Chilean datasets. These responses were broken down into phrases, allowing the identification of the primary themes. The first author then undertook the final analyses after the three evaluators compared results and reconciled any discrepancies.

### Statistical analysis

From the 716 participants that answered the survey, data from 15 participants with basic education level, 28 participants that did not buy eggs and 1 vegan were omitted from the study due to the low number within these categories. Descriptive statistics for the 672 usable responses were calculated using Microsoft Excel for Windows and all other statistical analyses were conducted using SAS 9.3. Consumption of animal products was re-organised into two categories: consume eggs, milk and all types of meat; and consume eggs and milk but not meat. Type of eggs usually bought was re-organised into three categories: regular; all types in different occasions; and specialty (free-range or organic). WTP ≥5% to buy eggs produced in the conditions described as important was re-organised into three categories: do not know; the same; and ≥5%.

There was no effect of country on the emerging themes, therefore data were combined and analysed together. The associations between emerging themes from the qualitative analysis and type of eggs and WTP ≥5% were calculated using the dataset from 530 participants due to the absence of a full dataset for 142 participants (83 participants that did not answer the question *Which type of eggs do you usually buy*? and 68 participants answered “do not know” for the question *Would you be willing to pay to buy eggs produced in the conditions that you described as important*?). Multinomial logistic regression (Proc Glimmix) was used to analyse associations of emerging themes with type of eggs participants usually buy and WTP ≥5% to buy eggs produced in the conditions that participants described as important. Emerging themes (Ethical issues; Animal welfare; Natural housing and feeding; Production) were considered a dependent variable and type of eggs participants usually buy and WTP ≥5% to buy eggs produced in the conditions that participants described as important were considered independent variables. Univariate models were built to separately assess the influence of each predictor variable on the dependent variables. The association between type of eggs participants usually buy and WTP ≥5% to buy eggs produced in the conditions that participants described as important was also analysed using PROC GLIMMIX. WTP ≥5% was considered as the dependent variable. Results are presented as odds ratio and 95% confidence interval (95% CI).

## Results

### Demographic data

Demographic data, separated by cohort, are presented in Table 1. Most participants had graduate or postgraduate degrees, lived in a city with more than 100,000 habitants, were not involved in animal production, consumed eggs, milk and all types of meat, and had internet as the main source of information regarding animal production systems, followed by general TV or radio programs in Brazil and friends in Chile.

**Table 1:** Demographic data (in percentage) of the cohorts (Brazilian participants [BR] and Chilean participants [CH]) that participated in the survey (n = 672).

Demographics	Variable		
		BR %	CH %
Sex			
	Female	51.8	54.4
	Male	48.2	45.6
Age (yr)			
	18–25	19.8	20.3
	26–35	29.9	32.3
	36–45	22.3	19.8
	46–55	14.3	12.5
	56–65	11.0	9.9
	>66	2.7	5.2
Level of education			
	High school graduate	31.7	17.7
	Graduate and postgraduate	68.3	82.3
Area of residence			
	City with < 20,000 habitants	8.2	8.7
	City between 20,001–100,000 habitants	16.8	3.2
	City between 100,001–1 million habitants	56.4	6.1
	City with > 1million habitants	18.6	82.0
Involvement in animal production			
	Not involved	91.2	84.0
	Involved	8.8	16.0
Consumption of animal products			
	Consume eggs, milk and all types of meat	97.6	96.8
	Consume eggs, milk but no meat	2.4	3.2
Knowledge about egg production systems			
	Completely unfamiliar	29.9	23.0
	A bit familiar	25.0	21.8
	Somewhat familiar	15.5	25.0
	A bit unfamiliar	24.4	19.5
	Very familiar	5.2	10.8
Source of information regarding animal production systems			
	Internet	57.6	54.1
	General programs	33.5	32.0
	Rural programs	33.2	11.9
	Friends	31.4	32.8
	Newspaper	19.8	23.8
	Scientific journals	10.4	13.4
	University	8.8	22.1
	ONGs	7.3	8.1
	Others	2.7	2.6

### Emerging themes of an ideal egg production farm and their reasons

Features of an ideal egg production farm and justifications were classified (Table 2) into seven different themes (and respective codes): animal welfare (freedom to move, space, innate behaviours, good feeding, shelter, happiness, health, not over-exploiting); natural housing or feeding (natural housing or living environment, natural feeding–low or without hormones, antibiotics or other chemical); egg quality and its effect on consumers’ health and nutrition (sensory quality, nutritional quality, healthy products); hygiene (cleanliness, organization, ventilation, air quality); production (profitability, production, efficiency, sustainability); ethical issues (respect, moral, ethics); and environment (environmental protection, odour, concerns about waste or animal manure). Many responses bridged more than one theme and were thus assigned into multiple themes. Many participants associated animal welfare and natural housing or feeding with egg quality and its effect on consumers’ health and nutrition. Results are described according to the frequency of themes for features and reasons covered by each cohort. In the qualitative analysis no differences were evident between the responses of Brazilian and Chilean respondents, and are therefore described together. Respondents are identified by country (BR, CH) and numbered from 1 to 328 for Brazilian and 1 to 344 for Chileans participants (e.g., BR 35 or CH 278).

**Table 2:** Emerging themes in responses by Brazilian (BR) and Chilean (CH) consumers to the questions, *What do you consider to be an ideal egg production farm*? and *Why do you consider these characteristics important*?

	BR (n = 328)		CH (n = 344)	
Main themes	n ^1^	% ^2^	n ^1^	% ^2^
Animal welfare	209	32.6	273	36.6
Natural housing or feeding	100	15.6	144	19.3
Egg quality and its effect on consumers’ health and nutrition	138	21.5	143	19.2
Hygiene	86	13.4	70	9.4
Production	52	8.1	46	6.2
Ethical issues	34	5.3	52	7.0
Environment	23	3.6	18	2.4
Total ^3^	642	100.0	746	100.0

^1^ Number of references classified into each theme.

^2^ Percentage of themes or reasons in relation to the total

^3^ Total number of themes or reasons identified in the responses. Note that a response could contain more than one theme or reason.

### Animal welfare

Animal welfare was considered important for many participants and they mentioned different farm characteristics that reflected a concern with the welfare of the hens [“Any type of farm where the hens have enough space to guarantee their welfare” (CH 138)]. The potential influence on the quality of life of the animals frequently justified the characteristics of the ideal farm [“For the welfare of the animals and, consequently, a production (system) free of suffering” (BR 59)].

Over 20% participants expressed a concern with freedom; comments often reflected participants’ concerns with space for the animals to move [“An ideal egg production farm would have space” (BR 93)]. Participants mentioned that hens should be free to move, in reference to the animal’s ability to perform some maintenance behaviours such as “walk”, “eat”, and “sleep” [“Space to walk and eat without stress” (BR 51)]. Participants also suggested that in the ideal egg production farm the animals should have a shelter for protection or that “during the day hens can go outside to walk, graze and rest under the shade” (CH 32). Hens’ emotional states were also mentioned: “Nobody can be happy if confined, even if it is an animal” (BR 7).

Participants also showed concerns with biological functioning, especially hens’ health, stating that hens must be healthy, without disease, and must receive veterinary care when necessary [“Space for all hens, well treated and veterinary attention in case of a problem” (CH 290)]. Other participants referred to care and the provision of good diets [“Well cared hens” (CH 277); “Very clean and appropriate diet for the hens” (BR 357)].

Participants also suggested that the quality of life of hens could affect their egg consumption and that “they refuse to be complicit in animal mistreatment applied by big companies, where the hens are immobile in tiny, crowded cages…” (CH 183).

### Natural housing and feeding

Natural housing and feeding was a salient characteristic identified in the statements of many participants, either in reference to the living environment [“A farm with enough space for the animals to feel in their natural habitat” (CH 351)]. Participants mentioned that animals should receive natural diets and showed negative attitudes towards the usage of hormones or antibiotics for egg production [“Without the use of hormones or other chemicals that accelerate the production process. Without genetic or hormonal interference” (CH 293)].

### Egg quality and effects on consumers’ health and nutrition

Another main aspect covered by participants was related to egg quality’s effects on consumers’ health and nutrition, “because it has an effect on consumers” (CH 237). Participants also mentioned concerns regarding the nutritional quality of the eggs, “as the nutrients of the eggs come from what the hens eat” (BR 310).

Some participants referred to the sensorial quality of the eggs: “I prefer “farm” eggs due to their colour and taste. The egg yolk is orange, and those with double yolk are spectacular (CH 21); “Because the treatment and diet offered to the hens affect the taste and the texture of the eggs” (CH 158).

Some participants associated hens’ welfare with egg quality [“Happy hens eating worms, because animal welfare is important for the quality of the eggs” (CH 352); “Free animals and with large spaces, as less stressed animals produce better products” (BR 73)]. They also seemed to perceive that animal welfare affects consumers’ health [“Outdoor system and without stress, because I believe that in this way we would eat high quality food” (CH 43)].

Finally, participants associated natural feeding (referring mostly, but not only to hens fed a diet without transgenic components, antibiotics, hormones, or other chemicals) with egg quality and effects on the consumer [“Natural feeding to avoid indirect hormone consumption” (BR 138); “A place where the hens receive the minimum amount of hormones and a less artificial diet because what we consume is directly associated with these substances, and they are not good for our health” (BR 1).

### Hygiene

Participants showed concern with the hygiene of the egg farm, especially related to farm facilities. Terms such as “clean”, “ventilated”, and “organized” were used to designate the ideal egg production farm. Some participants made reference to air quality, “dry and ventilated place” (BR 102).

### Production

Participants indicated that the ideal farm should be profitable, productive, efficient and sustainable [“A field where the hens have a large space to achieve high standard egg production” (CH 295); “A sustainable production system” (BR 169)]. In this context, the use of technology was praised by participants, where “highly technological can be used to reduce costs and increase profit” (BR 145).

Some participants associated productivity with animal welfare [“If the animals are living naturally and do not suffer stress, they will respond with higher productivity and with much more quality” (CH 163)].

### Ethical issues

Some reasons presented to justify the features of the ideal farm were ethical in nature, for example that the animals should be treated with respect and without cruelty or abuse. In some cases, participants presented a utilitarian view of the use of animals [“Because they are our food, and they are living beings” (CH 269).

### Environment

Participants showed concerns with the environmental impact of the egg production, such as water and manure treatment [“Organized, clean, where there is respect for the hens’ welfare and for the environment” (BR 252); “A good treatment of the water and the manure… so the industry will not severely impact the environment” (CH 184)].

### Closed questions

The frequencies of choices regarding the most important aspect in an egg production farm from the two cohorts were similar (Table 3). More than half of participants firstly chose the option *That this is the best for my health and for my family health* as the most important aspect in an egg production farm.

**Table 3:** Responses of the by Brazilian (BR) and Chilean (CH) consumers to the multiple-choice questions regarding the most important aspect in an egg production farm, the type of eggs they usually bought and willingness to pay more to buy eggs produced in the conditions that they described as important.

	BR (n = 328)		CH (n = 344)	
Questions	n ^1^	% ^2^	n ^1^	% ^2^
Which is the most important aspects in an egg production farm?				
That it is the best for my health and of my family	181	55.2	177	51.5
That it is the best for the welfare of laying hens	67	20.4	87	25.3
That it is the best for the environment	54	16.5	53	15.4
That it is the best for producing cheaper eggs	10	3.0	7	2.0
Other reasons	16	4.9	20	5.8
Which type of eggs do you usually buy? ^3^				
Regular	95	38.8	122	35.5
All types in different occasions	81	33.1	115	33.4
Specialty ^4^	69	28.2	107	31.1
In comparison with the price that you usually pay for eggs, how much more would you be willing to pay to buy eggs produced in the conditions that you described as important?				
The same	100	30.5	63	18.3
≥5%	186	56.7	255	74.1
Don’t know	42	12.8	26	7.6

^1^ Number of responses into each option.

^2^ Percentage of responses in relation to the total.

^3^ Eighty-three participants from Brazil did not answer “Which type of eggs do you usually buy?”, therefore the % was calculated using the total number of participants that answered this question (total = 245).

^4^ Specialty: free-range and organic eggs.

The frequencies of type of eggs participants usually bought, and of WTP more to buy eggs produced in the conditions that they described as important are presented in Table 3. Over 65% of participants answered that they would be WTP ≥5% to buy eggs produced in the conditions that they described as important in an ideal egg production farm.

### Associations between aspects considered important in an egg production farm, egg type purchasing habits and willingness to pay for eggs produced in an “ideal farm”

Participants that showed concerns for natural housing and feeding and the effects of egg quality on consumers’ health and nutrition had higher odds of buying specialty than regular eggs. In contrast, participants that were concerned with hygiene had lower odds of buying specialty than regular eggs (P≤ 0.05; Table 4). No association was found between concerns with animal welfare, production, ethical issues, or the environment and the type of egg participants bought (P> 0.05).

**Table 4:** The number and the ratio of participants that bought each type of eggs and mentioned each of the emerging themes (natural housing and feeding, egg quality and effect on consumers’ health and nutrition, and hygiene). Odds ratio and 95% confidence interval (95% CI) for binomial models of the type of egg participants usually buy and association with emerging themes are presented.

		Natural housing and feeding		Egg quality and effect on consumers’ health and nutrition		Hygiene	
	Total	n	Ratio	n	Ratio	n	Ratio
Regular	192	57	0.42	74	0.63	49	0.34
All types	157	53	0.51	64	0.69	44	0.39
Specialty	181	86	0.91	89	0.97	29	0.19
Outcome binomial models							
		Natural housing and feeding		Egg quality and effect on consumers’ health and nutrition		Hygiene	
		OR	95% CI	OR	95% CI	OR	95% CI
Regular							
All types		1.2	0.77–1.90	1.1	0.71–1.69	1.1	0.70–1.83
Specialty ^1^		2.1*	1.40–3.28	1.5*	1.02–2.33	0.6*	0.33–0.93

^1^ Specialty: free-range and organic eggs.

* Significantly different from reference category; P≤ 0.05.

OR = Odds ratios.

CI = 95% confidence interval.

Participants’ concerns with ethical issues, welfare, and natural housing and feeding were associated with higher odds of WTP ≥5% to buy eggs produced in the conditions that they described as important (P≤ 0.05; Table 5). In contrast, participants that mentioned aspects related to production had lower odds of being willing to pay ≥5% to buy eggs produced in the conditions that they described as important. No associations were found between concerns with egg quality and its effect on consumers’ health and nutrition, hygiene and environment and WTP more (P> 0.05).

**Table 5:** The number and the ratio of participants asked about their WTP ≥5% to buy eggs produced in the conditions described as important and mentioned each of the emerging themes: Ethical issues, welfare, natural housing and feeding and production. Odds ratio and 95% confidence interval (95% CI) for binomial models of the type of egg participants usually buy and association with emerging themes are presented.

		Ethical issues		Animal welfare		Natural housing and feeding		Production	
	Total	n	Ratio	n	Ratio	n	Ratio	n	Ratio
The same	145	6	0.04	81	1.27	42	0.41	32	0.28
≥5%	385	63	0.2	303	3.7	154	0.67	47	0.14
Outcome binomial models									
		Ethical issues		Animal welfare		Natural housing and feeding		Production	
		OR	95% CI	OR	95% CI	OR	95% CI	OR	95% CI
The same									
≥5%		4.5*	1.91–10.74	2.9*	1.94–4.40	1.6*	1.08–2.47	0.5*	0.30–0.81

* Significantly different from reference category; P≤ 0.05.

OR = Odds ratios.

CI = 95% confidence interval.

Participants that stated to be willing to pay ≥5% to buy eggs produced in the conditions described as important had higher odds of buying all types of eggs in different occasions and specialty eggs than regular eggs (P≤ 0.05; Table 6).

**Table 6:** The number and ratio of participants that bought regular, all types or specialty eggs, within those that were willing to pay ≥5% to buy eggs produced in the conditions described as important. Odds ratio and 95% confidence interval (95% CI) for binomial models of the type of eggs participants usually buy and association with those that pay ≥5% are presented.

	Willingness to pay more				
	Total	n	Ratio	OR	95% CI
Regular	192	123	1.78		
All types	157	119	3.13	1.76*	1.10–2.81
Specialty ^1^	181	143	3.76	2.11*	1.33–3.36

^1^ Specialty: free-range and organic eggs.

* Significantly different from reference category; P≤ 0.05.

OR = Odds ratios.

CI = 95% confidence interval.

## Discussion

To our knowledge, this study is the first to describe the views of Latin American consumers towards egg production systems, and to show associations between these views and egg purchasing habits. Participants expected a farm where laying hens are free to move and to perform innate behaviours, are healthy, are fed natural foods without excess additives, and are reared in a natural environment; often, as shown in other surveys [17, 28], participants linked these aspects with the sensory and nutritional quality of the product. For the majority of participants, human health should be the most important goal for an egg production farm, and many associated animal welfare and naturalness with higher egg quality and positive effects on human health and nutrition. To some extent, the views of participants towards an ideal egg production farm were associated with their type of egg purchasing habits and stated WTP a premium for specialty (free-range or organic) eggs. Many of the values and attitudes towards animal welfare expressed by Brazilian and Chilean participants support those described for citizens from other countries [17, 29].

Concerns with natural housing and feeding, with egg quality and a perceived effect on consumers’ health and nutrition were positively associated with buying specialty eggs. Additionally, participants that expressed concerns with hen welfare, with ethical treatment of the animals, and with naturalness of the production system showed more WTP to buy eggs produced in the conditions that met their expectations. Our findings are in accordance with studies from the UK [30], Spain [31], the US [32], and several European countries [33, 34], where respondents indicated WTP more for food produced under animal welfare standards, in cage-free systems, or under organic systems. As found in other studies [17, 35], the impact on consumers’ health was the most important attribute of a production system. Thus, WTP more for eggs produced under ethical conditions, with high animal welfare standards and by hens housed and fed naturally may be at least in part related to the perception that these aspects result in products of better quality, as stated by participants in this survey and others (see [17]). In this survey we used a direct approach to assess WTP, which is considered less robust than other methods, and may not be associated with real purchase behaviour [36]. A gap between attitudes towards animal production systems, stated willingness to pay for such products and purchasing behaviour is widely described [37]. Regarding farm animal welfare, although no important differences among countries have been identified [34], socio-demographic characteristics appear to account for most of the variation in WTP for farm animal welfare [25]. Thus, we encourage studies on WTP for alternative animal production systems in Latin America.

The consumption of free-range and organic eggs is growing in developed countries [38] and, although there are no reports in Brazil and Chile on trends in egg type consumption, a growth of demand can be inferred from growing availability of free-range and organic eggs in the market. However, as shown in other regions [38, 39], cost may represent a barrier for the production and consumption of specialty eggs in these countries. For example, a recent survey of a representative sample of Brazilians showed that 84% would like to purchase organic products, but did not due to price (62%) and difficulty to find organic products (32%) [40]. As in our study, European consumers declared themselves concerned with farm animal welfare [41] but less than 10% would pay a 10% premium for animal friendly products [42]. The partial associations between participants’ views and their purchasing habits and WTP indicate that many consumers may not be able or willing to purchase eggs with the qualities they consider important. In reference to this “citizen/consumer paradox”–where citizens appear to demand high farm animal welfare and environmental standards but usually buy the cheapest product, regardless of the production system—Aerts [43] concluded that “the food chain is not consumer-driven in the real sense of the word, nor is it producer driven: it is retail driven”. Indeed, retail driven changes are already occurring in egg production in Latin America, with restaurant chains and corporations committing to use only free-range eggs in their products [14]; these changes may not be a direct response to consumer and citizens demand, but possibly an anticipation of pressure from lobbies representing the interests of consumers or animal advocates [5].

Participants’ concerns with hens’ natural feeding, and their stated preference for feed containing no hormones, antibiotics or other chemicals is frequently reported, and as in our survey appear to be linked with the desire to consume safe, healthy products, and to avoid food that poses real or perceived risks to human health, to animal integrity and to the environment [17, 19, 23, 24, 44]. Perceived associations between natural production systems, hen welfare and egg quality were observed in this and in other surveys [32, 37]. Consumers’ belief in these relationships has also been described for other food animal products [23, 42], and may explain why naturalness is central to the public acceptability of production systems [17]. In disconnect with public preferences, and growing international calls for prudent use of antibiotics in animal production [45], industry stakeholders in Brazil defend the use of antibiotics as growth promoters or for prophylactic use as essential to achieve the production and economic goals of the industry (e.g. [46]).

In accordance with findings from other surveys [38, 47] some participants believed that free-range and organic eggs have higher nutritional value than regular eggs; a few also expressed beliefs that the quality of treatment and the diet offered to the hens would affect eggs’ taste and texture. This perception has not been supported by blind taste tests [39]; however, it has been shown that consumers’ sensory perceptions can be influenced by beliefs regarding the origin of beef [48]. A consumer misconception also appears to exist regarding differences in the nutritional value of eggs produced under different systems, as scientific evidence for such effect is restricted to nutrient-enhanced eggs [49].

The concerns with animal welfare shown by participants have been described for citizens from many countries, who value animal welfare and believe that conventional housing and management practices reduce hens’ welfare [32, 38, 39]. Participants mentioned animal welfare especially in relation to freedom to move, and naturalness in the context of housing and feeding; in their perception, animals have a better quality of life and can express innate behaviours in these conditions. Animals’ freedom to move to move and possibility to perform natural behaviours seemed to be a relevant concern for participants, as shown in other studies [17, 23, 50]. Participants also suggested that hens must be protected from suffering and stress, be healthy, and receive a good diet and a humane treatment. Altogether, this is in line with the concept of animal welfare proposed by Fraser et al [51] that covers aspects of the biological function, animal sentience and naturalness.

Abundant, affordable production, was a concern shown here and in other surveys [44, 52]. In contrast, some participants raised a criticism to production methods that they perceived to promote “unnaturally high” production rates. This issue, also identified by others [53, 54], is highly relevant in the current debate of sustainable animal production. Whereas some authors alert to some undesirable effects of genetic selection for high production on animal welfare [55, 56], others consider the use of highly efficient animals–in terms of feed conversion into animal protein–important to achieve the goal of minimising environmental impacts while producing enough animal protein to supply growing demands [57]. Although this issue was valued by some participants, the low priority participants invited to discuss egg farming gave to the environmental issue has also been shown on surveys of public views of cattle production systems [58].

### Some comments on the survey methodology

The convenience sample used in this survey does not represent the Brazilian or Chilean populations, as participants were arguably more urban and of higher socioeconomic status than the average of the countries’ populations. Through this recruiting, though, we were able to reach consumers of different egg types and prices, which allowed us to investigate associations between attitudes and purchasing behaviours. Thus, even if the results cannot be generalized to the general population of these countries, they contribute novel understanding of Brazilian and Chilean citizens’ knowledge, preferences, and perceptions regarding egg production systems.

The methodology applied in this survey was based on that used by Gaymard and Bordair [59], and adapted to investigate public views regarding dairy [58] and pig production [54]. In comparison with choice methodologies, where researchers choose topics on which participants will give their opinion [29, 32, 47], this methodology avoids soliciting participants’ opinion on issues they may have little or no understanding. We considered that relevant, given several reports of low awareness towards animal production systems and animal welfare among citizens in Latin America [5, 23, 60]. Social desirability bias (i.e. “people responding in a way that shows them in a good light” [61]) is a common limitation of studies exploring public attitudes, and may encompass both “self-deception (an honest but overly favourable self-image) and impression management (falsely presenting one’s self in an attempt to create a favourable impression)” [62]. Participants were recruited online or personally, but with minimal contact with the researcher, in an effort to reduce interviewer effects on social response bias [63]. However, the potential influence of social desirability bias needs to be considered when interpreting our results.

## Conclusions and animal welfare implications

Survey participants, egg consumers in two Latin American countries, often described more natural production systems, which they perceived to improve animal welfare and result in high egg quality, as ideal. The most cited features were hens’ freedom to move and to perform innate behaviours, natural housing and feeding, hygiene, and health standards. Importantly, participants’ views were associated with their egg purchasing choices and willingness to pay for eggs produced in such “ideal” system. These findings indicate a demand for more natural and animal friendly egg production systems and support for initiatives to ban restrictive conventional cage systems. Our results also indicate a disconnect between lay citizens’ expectations and industry practices, given that intensive confined systems, which predominate in this region, typically fail to supply many of these characteristics. Growing interest and involvement of the public in farm animal welfare is driving changes in egg production systems in Europe and North America; changes in available disposable income combined with increased access to information indicate that a similar trend may be expected in developing countries in the near future. To maintain or improve social sustainability of the egg industry, producers in the region may consider incorporating in the production systems some elements identified as relevant for consumers.
